# Decreasing glucocorticoid levels towards the expansion front suggest ongoing expansion in a terrestrial mammal

**DOI:** 10.1093/conphys/coab050

**Published:** 2021-07-02

**Authors:** Alexandre Azevedo, Liam Bailey, Victor Bandeira, Carlos Fonseca, Jella Wauters, Katarina Jewgenow

**Affiliations:** 1 Department of Reproduction Biology, Leibniz Institute for Zoo and Wildlife Research, Berlin, Germany; 2 Instituto de Ciências Biomédicas Abel Salazar - University of Porto, R. Jorge de Viterbo Ferreira 228, 4050-313 Porto, Portugal; 3 Department of Evolutionary Genetics, Leibniz Institute for Zoo and Wildlife Research, Berlin, Germany; 4 Department of Biology and CESAM, University of Aveiro, Campus Universitário de Santiago, 3810-193 Aveiro, Portugal; 5 ForestWISE - Collaborative Laboratory for Integrated Forest and Fire Management, Quinta de Prados, Campus da UTAD 5001-801 Vila Real, Portugal

**Keywords:** Egyptian mongoose, hair glucocorticoids, *Herpestes ichneumon*, range expansion, stress

## Abstract

Understanding the causes of range expansions in abundant species can help predict future species distributions. During range expansions, animals are exposed to novel environments and are required to cope with new and unpredictable stressors. Glucocorticoids (GCs) are mediators of the hormonal and behavioural mechanisms allowing animals to cope with unpredictable changes in the environment and are therefore expected to differ between populations at expansion edge and the historic range. However, to date, very few studies have evaluated the relationship between GCs and range expansion. The Egyptian mongoose has been rapidly expanding its range in Portugal over the past 30 years. In this study, we applied an information theoretic approach to determine the most important spatial and environmental predictors of hair GCs (hGCs) in the population, after controlling for normal patterns of hGC variation in the species. We observed a decrease in hGC as distance from the historic range increased (i.e. closer to the expansion front). This distance term was present in all of the top models and had a 95% confidence interval (95% CI) that did not overlap with zero, strongly supporting its influence on hGC. We estimated a 0.031 pg/mg (95% CI: −0.057, −0.004) decrease in hGCs for each kilometre distance to the Tagus River, which was once the limit of the species’ distribution. Our results indicate that the species’ expansion is unlikely to be limited by mechanisms related to or mediated by the physiological stress response. The decrease in hGC levels towards the expansion edge coupled with limited evidence of a negative effect of human population density suggests that the species’ northward expansion in Portugal could continue.

## Teaser

We determined spatial and environmental predictors of hair glucocorticoids (GCs) in an expanding population of Egyptian mongoose. There was strong support for a decrease in GC levels towards the expansion front, suggesting an ongoing expansion of the species.

## Introduction

The abundance, richness and spatial distribution of naturally occurring populations of wild species are changing at an unprecedented rate as a consequence of anthropogenic environmental change (Pimm *et al.*, 2014; Newbold *et al.*, 2015). Ecologists and evolutionary biologists have sought to understand the factors that shape species’ spatial distributions for decades (Holt, 2003; Liebl and Martin, 2012). In the Anthropocene, a small number of species have expanded their ranges, contradicting the general trend of decline driven by anthropogenic environmental change (Liebl and Martin, 2012). While the study of these exceptions can offer important information to predict how populations respond to environmental change, opportunities for such studies are rare (Hui *et al.*, 2012; Liebl and Martin, 2012, 2013).

Range expansions take place through the dispersal of individuals from the core population into novel environments. This is influenced by the set of environmental factors that enable the species to persist (niche), their spatial distribution (habitat) and the ability of the species to move (disperse) and adapt to new areas (Holt, 2003). Range expansions can take place when species occupy niche opportunities that arise due to changes in biotic or abiotic factors, such as changes in climate, land use or extirpation of competing species, or alternatively, due to the emigration of individuals from core populations into habitats where their niche requirements are not met, creating sink populations (Holt, 2003). The neuroendocrine stress response allows organisms to cope with unpredictable stressors in the environment (Wingfield *et al.*, 1998). Accordingly, the colonization of new and unfamiliar environments during range expansions is expected to be facilitated by increased stress reactivity (Liebl and Martin, 2012; Martin *et al.*, 2017). However, if the new and unknown environment leads to very frequent or chronic activation of the stress response, impacts of elevated glucocorticoids (GCs) on survival, reproduction and fitness may occur (Sapolsky *et al.*, 2000; Bonier *et al.*, 2009a).

In vertebrates, GCs are released by activation of the hypothalamic–pituitary–adrenal axis (HPA-axis) in response to challenging environmental stimuli (Wingfield *et al.*, 1998; Sapolsky *et al.*, 2000). This neuroendocrine response allows animals to respond to environmental cues (Wingfield and Mukai, 2009) and adjust their physiology and behaviour to cope with and recover from unpredictable environmental change (Wingfield *et al.*, 1998; Sapolsky *et al.*, 2000; Zimmer *et al.*, 2020). Due to the pervasive effects of GCs, chronic elevations are thought to result in deleterious effects on survival, reproduction (Sapolsky *et al.*, 2000) and fitness (Breuner *et al.*, 2008; Vitousek *et al.*, 2018). However, relations between baseline and stress-induced blood and faecal GC levels and fitness have been inconsistent (Bonier *et al.*, 2009a, 2009b; Dantzer *et al.*, 2014) and context dependent (Creel *et al.*, 2013; Vitousek *et al.*, 2018).

Differences in HPA-axis physiology could influence the ability of animals to colonize new environments during range expansion, but studies evaluating this link are scarce and focus on birds, reptiles and amphibians (e.g. Atwell *et al.*, 2012; Liebl and Martin, 2012; Brown *et al.*, 2015; Martin *et al.*, 2018). For example, in expanding populations of house sparrows (*Passer domesticus*), individuals at the range edge have been shown to exhibit increased stress-induced GC levels and differences in the expression of the receptors involved in GC pathways, which is thought to facilitate their ability to colonize novel environments (Liebl and Martin 2012, 2013; Martin *et al.*, 2017). In dark-eyed juncos (*Junco hyemalis*) and cane toads (*Rhinella marina*), a difference in GC reactivity was also observed between individuals of the newly established and historic populations, but with the colonists or edge populations showing decreased stress-induced GC levels (Atwell *et al.*, 2012; Brown *et al.*, 2015).

Baseline levels of GCs during range expansions have also shown inconsistent trends (Atwell *et al.*, 2012; Liebl and Martin, 2012; Martin *et al.*, 2017). A broad scale study including approximately one hundred species of birds and reptiles found little evidence of a relation between stress-induced or baseline GCs and edge/non-edge location within the population (Martin *et al.*, 2018). The inconsistency of these findings could be due to methodological factors, such as the lability of point samples of blood GC measurements (Bonier *et al.*, 2009a). Baseline GCs based on plasma samples have shown low intra-individual repeatability (Vitousek *et al.*, 2018, 2019b) compared to GC measurements from matrices reflecting long-term GC levels (Taff *et al.*, 2018) and are greatly influenced by environmental conditions. Alternatively, these inconsistencies could be explained by the context dependence of stress-induced and basline levels of GC (Vitousek *et al.*, 2018) or the fact that they are simply separate traits shaped by different selective pressures (Vitousek *et al.*, 2019a).

Hair GC (hGC) measurements are thought to reflect both baseline and stress-induced GCs incorporated into hair over prolonged periods (D’Anna-Hernandez *et al.*, 2011; Kapoor *et al.*, 2018; Short *et al.*, 2016) and suffer little influence from short-term variations such as those caused by capture or handling. This may allow the identification of long-term trends in overall GC exposure that would be difficult to detect using matrices reflecting short-term variations. For example, a wild population of red deer (*Cervus elaphus*) exhibited an increase in hGC levels in response to hunting activity that was not detectable in faeces, while baseline plasma GC levels actually tended to decrease (Vilela *et al.*, 2020). Hence, information on long-term GC exposure obtained from hGC analysis could help understand range expansions and predict whether expanding populations are likely to become established and pose an invasion risk (Martin *et al.*, 2017) or result in sink populations.

**Figure 1:**
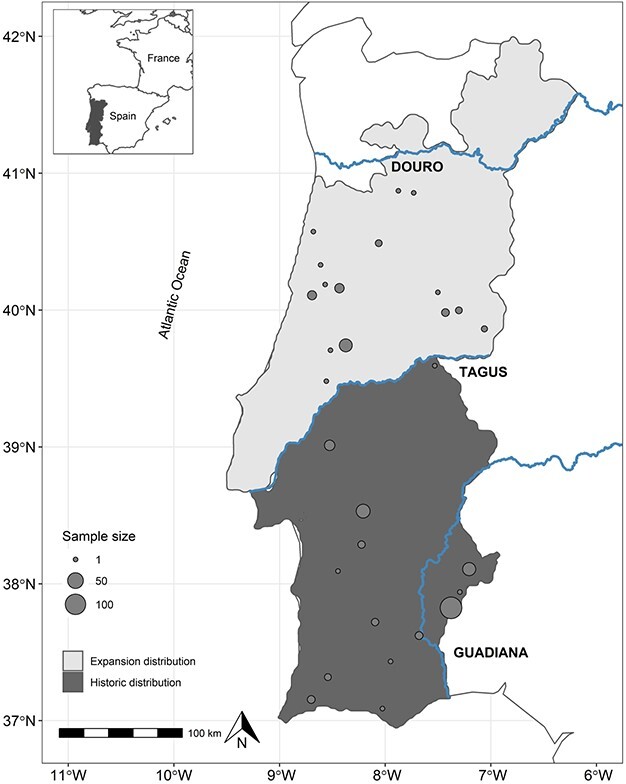
Geographic distribution of the Egyptian mongoose in Portugal. The species was confined to the South of the Tagus River (dark grey area). In the past three decades, it has been rapidly expanding northward (light grey area). Circles represent number of specimens sampled in each location.

The Egyptian mongoose (*Herpestes ichneumon*) is a medium sized carnivore that is widely distributed across Africa and the Middle East. In Europe, it is only present in the Iberian Peninsula, most likely due to colonization in the Late Pleistocene (Gaubert *et al.*, 2011). In the past three decades, the species has rapidly increased its range to the north of the Tagus River (Fig. 1), which was once considered a natural barrier to its expansion (Barros *et al.*, 2016b). In the expansion area, the species experiences very different environmental conditions, such as higher human density and primary productivity, and lower availability of favourable habitat (Bandeira *et al.*, 2016). Based on presence–absence data, changes in land use such as rural abandonment have been identified as drivers of the expansion of the Egyptian mongoose (Barros *et al.*, 2015). Morphological differences have been identified between the populations inhabiting the historic and expansion areas, with lower size (Bandeira *et al.*, 2016) and body condition (Bandeira *et al.*, 2019) and higher testicular mass (Bandeira *et al.*, 2021) in the expansion area. However, no information exists on GC levels across the species’ range.

In order to be informative, GC measures require species-specific validation (Touma and Palme, 2005; Azevedo *et al.*, 2020; Jewgenow *et al.*, 2020) as well as consideration of how ‘normal’ patterns of variation (such as age, sex and season) and interacting environmental factors influence the physiological response (Dantzer *et al.*, 2014). Both cortisol and cortisone are stress hormones that have been identified in Egyptian mongoose hair (Jewgenow *et al.*, 2020). In previous work, we cross-validated an enzyme immunoassay (EIA) targeting cortisol and cortisone in guard hairs of this species with liquid chromatography coupled with mass spectrometry (LC–MS/MS) and high-pressure liquid chromatography (HPLC) (Jewgenow *et al.*, 2020) and characterized normative variations with age, sex and season within the free-ranging population inhabiting Portugal (Azevedo *et al.*, 2019).

In the current study, we measured hGC in Egyptian mongoose over the species’ entire range within Portugal. We applied an information theoretic approach (Burnham and Anderson, 1998) to determine which spatial (historic vs. expansion region, distance to Tagus river within the expansion population) and environmental factors [area of favourable habitat, European rabbit (*Oryctolagus cuniculus*) harvest data, Egyptian mongoose harvest data, human population density] from our data set influence hGC levels in the population, while controlling for known effects of age, sex, season and sample storage time. To our knowledge, this is the first study assessing the relation of GC with a mammalian range expansion and the only study using integumentary long-term GC measurements. If higher long-term GC levels facilitate expansion just as stress-induced blood GC levels seem to (Liebl and Martin, 2012, 2013; Martin *et al.*, 2017), we expect to see higher hGC levels in the expansion area, with tendency to increase as the distance to the Tagus river increases. Alternatively, a decrease in hGC levels towards the expansion front would be more likely in a scenario where animals in the expansion area were presented with less frequent or severe stressors or where lower baseline hGCs favoured colonization.

## Methods

### Sample collection

We obtained data and hair samples from 294 carcasses of wild Egyptian mongoose collected throughout the year, between January 2008 and December 2014 from hunting activities throughout mainland Portugal (Bandeira *et al.*, 2016). After the exclusion of specimens for which data were missing, 236 samples remained and were used in this study. Carcasses were stored frozen at −20°C and then thawed at the time of sample collection. Hair was clipped with scissors as close to the skin as possible from a standard location (between the ‘scapulae’) in order to account for variation in hGC with anatomical region (Azevedo *et al.*, 2019). Hair samples were stored in paper envelopes in a dry and dark location until the date of GC extraction.

### hGC measurement

hGCs were quantified by an EIA for which validation was previously performed (Azevedo *et al.*, 2019). Briefly, 20 mg of guard hairs were separated from undercoat, washed twice with 90% methanol for 5–10 seconds and dried at 70°C. Next, 10 mg of hair were weighed and ground to a fine powder in a Precellys24 tissue homogenizer (Bertin Technologies, France). Finally, GCs were extracted from the powdered hair with 90% methanol and centrifuged, and the supernatant was collected and frozen until the day of GC measurement. hGCs were measured with an EIA using a polyclonal antibody (rabbit) against cortisol-3-CMO-BSA and cortisol-3-CMO-peroxidase as label. The assay was validated by demonstrating parallelism of serially diluted hair extracts to the cortisol standard curve, and the inter-assay coefficients of variation were 10.78% for extracts containing low and 15.95% for extracts containing high concentrations of cortisol. The intra-assay coefficients of variation were 6.72% for extracts containing low and 5.37% for extracts containing high concentrations of cortisol and the sensitivity of the assay was 0.40 pg/well. In order to determine if the EIA was targeting the intended steroids, it was validated by HPLC analysis that demonstrated that the cortisol-3CMO antibody was binding to cortisol and small amounts of cortisone, and finally by demonstrating a strong correlation between cortisol-3CMO-EIA measurements and HPLC-MS/MS measurements of cortisol and cortisone from the same extracts. The validation of the EIA is reported in detail in Azevedo *et al.* (2019).

### Spatial variation

In order to assess how expansion is related to hGC levels, Egyptian mongoose specimens were assigned to the ‘historic’ region if they were captured south of the Tagus River, or to the ‘expansion’ region if they were captured north of the Tagus River (Fig. 1). For specimens captured in the ‘expansion’ region, the shortest distance from capture location to the Tagus River in kilometres was calculated, and the resulting variable ‘distance to river’ was included in model construction to assess whether being closer to the expansion edge (or further from the core population) influenced hGC levels.

### Normative patterns of variation

In our previous study age, sex and storage time were shown to influence hGC measurements in this species (Azevedo *et al.*, 2019). hGC levels were higher in males compared to females and in juveniles younger than 5.5 months compared to other age cohorts and decreased with storage time (Azevedo *et al.*, 2019). Therefore, the effect of these variables was accounted for by including them in the model. Each mongoose was classified as an adult (over 1 year of age), sub-adult (between 9 and 12 months), type 2 juvenile (between 5.5 and 9 months) and type 1 juvenile (between 2.5 and 5.5 months of age) based on dental development (Bandeira *et al.*, 2016). Specimens were designated as male or female based on the presence of testicles or ovaries. Storage time was defined as the total number of days between the date of capture of the mongoose and the date of cortisol extraction from hair (1150 to 2266 days). Although not a significant factor in our prior analyses (Azevedo *et al.*, 2019), seasonal variations in GCs have often been demonstrated in vertebrates (Romero, 2002). Season was included in our model to account for the species’ seasonal reproductive activity with a peak in spring, which is possibly delayed in the expansion region (Bandeira *et al.*, 2021). Animals were assigned to winter (January to March), spring (April to June), summer (July to September) or autumn (October to December) according to date of capture. We included snout–tail length (STL) values obtained by standard mammal measurement methods to account for the potential effect of metabolic rate on baseline GCs, and because smaller animals may have less energy reserves and hence require enhanced GC responsiveness to meet unpredictable energy demands (Haase *et al.*, 2016; Francis *et al.*, 2018; Vitousek *et al.*, 2019a). Finally, we included an index of body condition score (BCS) to account for the amount of energy reserves present in each specimen at the time of capture. We expect body condition to influence GC levels differently from size because of the central role of GCs in the regulation of energy metabolism (Sapolsky *et al.*, 2000). For calculation of the BCS, we used the ‘scaled mass index’ based on body mass scaled for STL (Peig and Green, 2009, 2010).

### Environmental factors

The environmental factors used for model construction were selected based on our predictions of their biological relevance for hGC measurement drawn from results of previous studies with the species (Barros *et al.*, 2015, 2016a; Bandeira *et al.*, 2016, 2018, 2019). All environmental variables were presented as mean values within the 2 × 2 km grid cell where the specimen was collected. The reported home range size for the Egyptian mongoose in the Iberian Peninsula is 3.10 ± 2.12 km^2^ (Palomares, 1994). Hence, the grid cell area of 4 km^2^ is likely to offer an approximation of the environmental conditions each specimen experiences in its territory. We used the area occupied by shrub and/or agro-forestry habitat in each grid cell to obtain a proxy of the availability of habitat types that have been shown to favour gene flow (Barros *et al.*, 2016a) and expansion (Barros *et al.*, 2015) of the species in Portugal. Our prediction was that habitat types that have been favourable to the species’ expansion would be associated with lower hGC levels. Human population density presented as the number of inhabitants per km^2^ in each grid cell (data from Eurostat) (European Commission, 2015) was included as a fixed factor because increased levels of GCs are generally observed with increasing human disturbance (Dantzer *et al.*, 2014). The extent of road network represented by the total length of road in metres in each grid cell (IGP, Instituto Geográfico Português, 2015) was included as a candidate factor in the model but was excluded due to collinearity with human population density. We included the number of Egyptian mongoose reported from hunting bags for each grid cell in the year and month (ICNF, unpublished data) of each specimen’s capture as a proxy of relative abundance of conspecifics to account for the effect of mongoose density on GCs. Population density can influence circulating GC levels in vertebrates, especially in non-social territorial species like the Egyptian mongoose where the frequency of social interactions at higher densities leads to more frequent activation of the HPA-axis (Creel *et al.*, 2013). Similarly, European rabbit (*O. cuniculus*) yields for each grid cell in the respective year and month (ICNF, unpublished data) were used as a proxy of relative prey availability because food scarcity or unpredictability may influence GC levels directly (Fokidis *et al.*, 2012).

### Statistical methods

We analysed the effect of spatial and environmental factors on Egyptian mongoose hGC using linear mixed effects models with a Gaussian error distribution. Input variables were standardized on two standard deviations to account for differences in scale and to enable comparison of effect sizes (Gelman, 2008; Schielzeth, 2010). Variance inflation factors (VIFs) were used to test for multi-collinearity between variables with a cut-off value of 4. Collinearity was detected between road network (VIF = 6.50) and human population density (VIF = 5.16), which were highly correlated (*r*_(234)_ = 0.86, *P* < 0.001). We considered the latter a more robust measure of human presence, as it is likely to include the effect of road network and many other factors. Therefore, road network was excluded from further analyses. The global model included the effect of the fixed factors age, sex, season STL, BCS, storage time, region, distance to river, favourable habitat, human population density, relative prey availability and relative conspecific density on hGC concentration. We also included the interactions between STL and both sex and age to account for differing effects of body size according to age cohort or sex. Year of capture was included as a random factor to account for non-independence and differences in GC levels in different years. Residuals of the fitted model were visually inspected by plotting against fitted values and with a Q-Q plot, to check model assumptions. We identified and removed two outlier hGC values that were more than six standard deviations from the mean and were causing violations in assumptions of homoscedasticity and normality of residuals. We cannot rule out the possibility that these values are the result of severe stressors, since 4-fold increases in hair cortisol have previously been documented (del Rosario *et al.*, 2011; Mastromonaco *et al.*, 2014). However, we did not consider the effect of these potentially severe and rare stressors useful to answer our current research questions. After outlier removal, residuals of the fitted model displayed an approximately normal distribution with no strong pattern of over-dispersion or heteroscedasticity. The candidate model set included 6656 models that were ranked based on AICc (ΔAICc from the best model ≤ 2.0) (Burnham and Anderson, 1998). We determined the relative importance of each factor using the sum of Akaike weights (*sw*) in the entire candidate model set, with 1 being the most important and 0 the least important. Factors that appeared in a higher number of models from the top model set and had higher sum of weights were considered more likely to be contained in the model best approximating the truth. We performed model averaging (Burnham and Anderson, 2002; Lukacs *et al.*, 2010) on the top model set (ΔAICc ≤ 2.0; Grueber *et al.*, 2011) to account for uncertainty in model selection and obtain more robust estimates. Statistical analyses were performed using the R statistical system v 4.0.3 (R Core Team, 2020); model selection for mixed models was conducted using ‘lme4’ package (Bates *et al.*, 2015) and ‘MuMIn’ package for model selection (Barton, 2020).

**Table 1:** hGC values (mean ± SD in pg/mg) and number of Egyptian mongoose specimens from each region, age cohort and sex included in statistical analyses

	Historic region 19.03 ± 5.68(n = 173)		Expansion region 18.83 ± 4.65(n = 61)	
	Female 18.44 ± 4.28 (n = 91)	Male 19.68 ± 6.88 (n = 82)	Female 18.01 ± 4.39 (n = 34)	Male 19.84 ± 4.84 (n = 27)
Adult 17.78 ± 4.74 (n = 141)	18.49 ± 4.26 (n = 56)	19.16 ± 5.47 (n = 46)	17.84 ± 4.75 (n = 24)	20.23 ± 3.96 (n = 15)
Sub-adult 19.42 ± 6.35 (n = 24)	17.1 ± 2.4 (n = 10)	24.97 ± 9.47 (n = 7)	16.38 ± 2.11 (n = 4)	18.24 ± 2.45 (n = 3)
Juvenile 2 16.99 ± 4.83 (n = 39)	16.77 ± 4.12 (n = 11)	16.62 ± 4.48 (n = 20)	18.5 ± 2.94 (n = 4)	17.96 ± 9.85 (n = 4)
Juvenile 1 22.11 ± 7.02 (n = 30)	20.53 ± 4.9 (n = 14)	25.07 ± 10.65 (n = 9)	22.32 ± 5.35 (n = 2)	21.15 ± 3.7 (n = 5)

## Results

Data for a total of 234 specimens were included in the analysis (Table 1). Among these, 141 belonged to the adult cohort, 24 to the sub-adult, 39 to juvenile 2 and 30 to juvenile 1, with a balanced distribution of females (125) and males (109). A total of 173 specimens were captured in the historic region and 61 were captured in the expansion area. hGC levels in Egyptian mongoose hair had a mean of 18.98 ± 5.42 pg/mg and varied between 8.07 and 43.36 pg/mg.

Model selection resulted in 6656 candidate models (Table S1), with a set of 16 models with ΔAICc ≤ 2.0 (Table 2). The distance to the Tagus River for specimens collected in the expansion area appeared in all of the top models and had a relative importance of 0.69 (Table 3). In terms of environmental variables, relative mongoose and rabbit availability were present in most of the top models and had moderate relative importance based on sum of Akaike weights. There was little evidence in support of an effect of human population density, relative area of favourable habitat and region on hGC levels. Age, sex, body condition, body size and sample storage time appeared in all or the majority of the top models and had sums of Akaike weights higher than 0.60, providing evidence for their influence on hCG levels.

**Figure 2:**
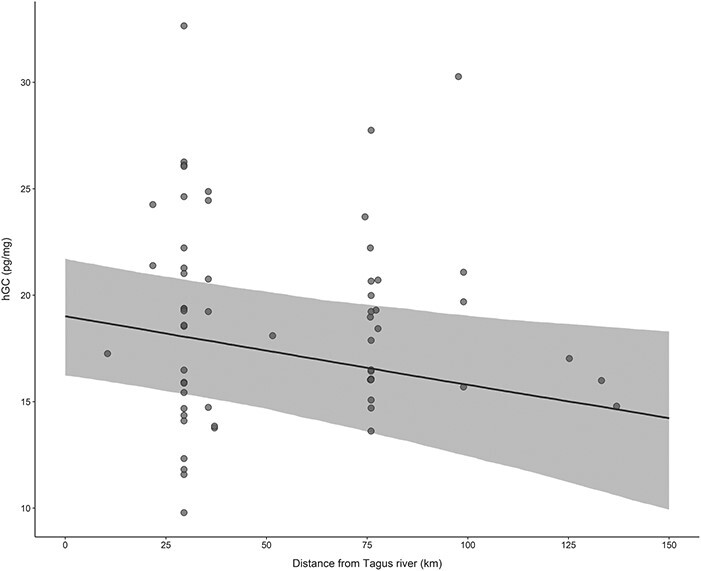
Model predictions of the effect of distance from the historic range (limited by the Tagus River) on hGC levels in the Egyptian mongoose. The plot shows model predictions and 95% CI (grey band) based on parametric bootstrapping with 5000 iterations.

Based on the estimates obtained by model averaging using untransformed data (Table 4), hGCs were estimated to decrease 0.031 pg/mg (95% CI: −0.057, −0.004) for each 1 kilometre increase in distance from the capture location to the Tagus River, in the expansion area (Fig. 2). In terms of environmental factors, hGCs were predicted to decrease 0.258 pg/mg (95% CI: −0.513, −0.002) for each additional mongoose harvested in the 2 × 2 km grid cell during that month and to increase 0.011 pg/mg (95% CI: 0.000, 0.023) with each harvested rabbit, after accounting for storage time and normative patterns of variation in the species.

Model averaged effect sizes within the top models (ΔAICc ≤ 2.0; Table 4) indicated that STL had the strongest effect on hGC concentration (−5.20), followed by age (−4.23, juvenile type 2), sample storage time (3.65), sex (2.08, male), distance to the Tagus River within the expansion area (−1.76) and BCS (−1.76). The effects of the number of harvested mongoose (−1.35) and rabbits (1.19), as well as season (−1.43, summer) and human population density (1.22), were weak when compared to the other factors. Interaction of age and sex with STL, region and favourable habitat were not present in the top model set.

**Table 2:** Model selection table; top ranked models with ΔAICc ≤ 2.0 are shown here (for full model selection table see Table S1); *R*^2^ and adjusted *R*^2^ (Adj. *R*^2^) values for each model are included (Nakagawa and Schielzeth, 2013); *w* indicates model weights

Age	Region	Sex	Season	Mongoose	Rabbit	BCS	Habitat	Population	STL	Storage	Age:STL	D. river	Sex:STL	R^2^	Adj.R^2^	df	AICc	ΔAICc	*w*
+	−	2.10	+	−1.14	1.27	−1.86	−	1.09	−5.34	−3.78	−	+	−	0.33	0.33	17.00	1397.29	0.00	0.017
+	−	2.11	+	−1.44	1.28	−1.91	−	−	−5.62	−3.64	−	+	−	0.32	0.32	16.00	1397.34	0.05	0.016
+	−	2.09	+	−1.20	1.23	−1.77	−	1.09	−5.05	−3.84	−	+	−1.74	0.34	0.34	18.00	1397.50	0.22	0.015
+	−	2.10	+	−1.50	1.24	−1.81	−	−	−5.33	−3.70	−	+	−1.74	0.33	0.33	17.00	1397.56	0.27	0.015
+	−	2.01	+	−	1.18	−1.73	−	1.43	−5.17	−3.94	−	+	−	0.32	0.32	16.00	1397.84	0.55	0.013
+	−	2.00	+	−	1.14	−1.64	−	1.45	−4.89	−4.00	−	+	−1.61	0.33	0.33	17.00	1398.36	1.07	0.010
+	−	2.17	−	−1.47	1.08	−1.58	−	1.16	−5.42	−	−	+	−	0.30	0.30	13.00	1398.82	1.53	0.008
+	−	2.00	+	−1.10	−	−1.77	−	1.11	−4.67	−3.93	−	+	−1.84	0.33	0.33	17.00	1399.05	1.76	0.007
+	−	2.01	+	−1.03	−	−1.87	−	1.11	−4.96	−3.87	−	+	−	0.32	0.32	16.00	1399.07	1.79	0.007
+	−	1.94	+	−	−	−1.75	−	1.42	−4.83	−4.01	−	+	−	0.31	0.31	15.00	1399.09	1.80	0.007
+	−	2.16	−	−1.52	1.06	−1.49	−	1.15	−5.15	−	−	+	−1.67	0.30	0.31	14.00	1399.14	1.85	0.007
+	−	2.01	+	−1.41	−	−1.82	−	−	−4.95	−3.79	−	+	−1.84	0.32	0.32	16.00	1399.20	1.91	0.006
+	−	2.02	+	−1.34	−	−1.92	−	−	−5.25	−3.73	−	+	−	0.31	0.31	15.00	1399.21	1.93	0.006
+	−	2.20	−	−1.55	1.02	−1.57	−	1.23	−5.26	−1.96	−	+	−	0.30	0.30	14.00	1399.25	1.96	0.006
+	−	2.18	−	−1.61	1.00	−1.47	−	1.23	−4.95	−2.09	−	+	−1.81	0.31	0.31	15.00	1399.25	1.96	0.006
+	−0.78	2.07	+	−1.43	1.26	−1.88	−	−	−5.65	−3.67	−	+	−	0.32	0.33	17.00	1399.29	2.00	0.006

Legend: BCS, body condition score; STL, snout–tail length; Age:STL, interaction between age and snout–tail length; D. river, distance to the Tagus River for animals captured in the expansion region; sex:STL, interaction between sex and snout–tail length; df, degrees of freedom; AICc, Akaike information criteria; ΔAICc, difference in AICc to the model with lowest AICc; w, Akaike weight. ‘+’ indicates a categorical variable that was included in the model, while ‘−’ indicates a variable that was not present.

**Table 3:** Relative importance of predictors based on the sum of Akaike weights in the complete set of 6656 candidate models

	Sum of weights	N containing models
STL	1.00	4608
Sex	0.99	4096
Age	0.98	4096
BCS	0.84	3328
Mongoose	0.72	3328
D. river	0.69	3328
Storage	0.63	3328
Season	0.61	3328
Rabbit	0.60	3328
Population	0.52	3328
Sex:STL	0.48	1536
Region	0.34	3328
Habitat	0.29	3328
Age:STL	0.13	1536

Legend: BCS, body condition score; STL, snout–tail length; Age:STL, interaction between age and snout–tail length; D. river, distance to the Tagus River for animals captured in the expansion region.

## Discussion

In this study, we aimed to determine which spatial and environmental factors influence long-term adrenocortical activity in the Egyptian mongoose population in Portugal, in order to better understand its idiosyncratic expansion in the context of anthropogenic change.

We found support for a relation of hGC levels with range expansion in the Egyptian mongoose. The distance between capture location and the Tagus River, in animals from the expansion area, appeared in all models with ΔAICc ≤ 2.0 and had a sum of Akaike weights of 0.69, strongly supporting its inclusion in the model best approximating the truth. Based on the averaged estimates of the top 16 models using standardized input variables, the effect size of the distance to the Tagus (−1.76) was of a magnitude comparable to the effect of sex (2.08) or body condition (−1.76) with a 95% confidence interval that did not include zero (−3.26, −0.26). hGC levels are estimated to decrease 0.031 pg/mg per kilometre as the distance from the Tagus River (and historic distribution) increased, which would equate to an estimated 1.0 pg/mg decrease in hGC every 32.26 km (Fig. 2). The estimated decrease in hGCs from the Tagus River to the expansion front (136.95 km) equates to 22% (4.24 pg/mg) of the mean hGC levels for the population (18.98 ± 5.42 pg/mg). These values were obtained while accounting for hGC variation with age, sex, season, sample storage time, body condition (BCS), size (STL) and environmental factors (conspecific and prey availability, human population density and relative area of favourable habitat). We found no evidence to support an effect of region (expansion vs. historic) on hGC levels. The effect of the distance to the Tagus River is influenced by three samples collected at distances above 125 km (Fig. 2), which, in case they were somehow related, would raise concern about possible confounding effects. Inspection of the data on the three samples revealed they were collected at different locations, in different years and seasons, by different people. Additionally, the effect of the distance to the historic region in an analysis performed without these three specimens resulted in qualitatively similar results.

It is not possible to discern whether our results reflect a phenotypic difference in long-term GC levels facilitating expansion or reduced GC exposure due to an environment with less frequent or severe stressors we could not account for (e.g. predators or competing carnivores). Decreasing GCs towards the expansion edge could reflect lower energy requirements to maintain physiological balance (McEwen and Wingfield, 2003), individuals in better condition and facing less challenges, potentially resulting in increased fitness (Bonier *et al.*, 2009a) or a reduced likelihood to exceed the normal and non-pathological response to environmental challenges (Romero *et al.*, 2009). The decreasing hGC levels towards the expansion front suggest that the Egyptian mongooses’ expansion is not likely to be limited by mechanisms related to or mediated by the physiological stress response. The negative association between hGC and distance to the historic range is consistent with the colonization of a new area where stressors are less frequent or less severe (e.g. niche opportunity or forced dispersal), as well as a scenario where lower baseline GC levels favour dispersal or survival at the expansion front.

Our results apparently contradict the findings linking increased stress-induced GCs with expansion in house sparrows (Liebl and Martin, 2012). However, while stress-induced GC measurements in sparrows reflect the reactivity of the HPA-axis, long-term hGC levels are thought to reflect circulating GCs over several weeks. The latter method is inadequate to assess phenotypic differences in HPA-axis reactivity favouring dispersal and survival in novel environments. However, it is likely to provide a better indicator of chronic GC exposure, which is thought to reflect the extent of environmental challenge to homeostasis (McEwen and Wingfield, 2003; Romero *et al.*, 2009) and to potentially influence fitness through the effects of chronically elevated GC levels on most peripheral tissues (Sapolsky *et al.*, 2000). Different response patterns and trade-offs between the effects of baseline and stress-induced GCs on fitness could explain simultaneously low baseline and increased stress-induced GC in expanding populations (Vitousek *et al.*, 2018, 2019a). Due to the existence of a (albeit dynamic) threshold above which GCs cause deleterious effects to the organism, it is expected that lower baseline levels are required to allow higher levels of stress-induced increases without reaching pathological levels of GC exposure.

Body size (STL), sex, age and body condition (BCS) were included in all of the 16 models with ΔAICc ≤ 2.0, providing strong support for their inclusion in the model explaining hGC variation. Based on the sum of Akaike weights, STL had the highest relative importance (1.00), followed by sex (0.99), age (0.98) and finally BCS (0.84). Regarding the effect of sex on hGCs, the results of this study are consistent with our prior research, with males presenting higher GC levels compared to females (Azevedo *et al.*, 2019). However, the variation of hGC among age cohorts is strikingly different from our previous work, where only type 1 juveniles differed from other cohorts, exhibiting the highest hGC levels. Here, with the inclusion variables reflecting body size and BCS, adults presented the highest hGC levels, followed by sub-adults, type 1 juveniles and finally type 2 juveniles. These results suggest that increased GC levels observed in juveniles could be at least partially driven by metabolic scaling or energy availability rather than exclusively by ontogenetic variation in endocrine mechanisms. Body size received the most support for inclusion in the model and had the strongest effect (−5.20, 95% CI: −7.56, −2.84) on hGCs that decreased with size. Energy reserves represented by BCS also received strong support for inclusion in the model and had a strong effect (−1.76, 95% CI: −3.09, −0.43), with hGC levels decreasing as body condition increased. Overall, the effects of variables accounting for normal patterns of hGC variation in this population were quite strong (with magnitudes ranging from 1.76 to 5.20) compared to the effect of spatial and environmental factors. Additionally, storage time was present in 14 of the 16 models with ΔAICc ≤ 2.0, with a sum of Akaike weights of 0.63 and a standardized effect of −3.65 (95% CI: −5.40, −1.90), supporting its inclusion in the model. These results illustrate how failing to include known causes of GC variation could confound the results of studies aiming to investigate the effects of environmental or spatial factors.

Human population density was expected to be associated with increased hGCs. However, we did not find strong support for its inclusion in the model (present in 11 of 16 models with ΔAICc ≤ 2.0, *sw =* 0.52 and 95% CI: −0.18, 2.62), questioning whether the species is severely stressed by human presence. Resilience to stress caused by human presence due to the species’ known behavioural plasticity (Monterroso *et al.*, 2014; Streicher *et al.*, 2020) could have facilitated expansion in spite of increasing human population density. Alternatively, an attenuation of the stress response due to habituation (Cyr and Romero, 2009; Dickens and Romero, 2013) could explain absence of an increase in hGC levels with human density. Nevertheless, the result is discordant with the general trend in vertebrates, where an increase in GC levels is usually observed with increasing human disturbance (Dantzer *et al.*, 2014). In the specific case of the Egyptian mongoose, presence–absence data previously revealed a negative influence of urban areas and human infrastructure on the species’ occurrence (Barros *et al.*, 2015). However, the absolute values of human density in our data were quite low, with a mean (and inter-quartile range) of human population data of 1(1–44) inhabitants per km^2^, compared to the country’s average of 112.5 (0–5244.6) inhabitants per km^2^ (from Eurostat, European Commission, 2015) Therefore, although our results suggest little influence of human presence on Egyptian mongoose hGCs at these human population densities, an effect might be present at higher densities, warranting cautious interpretation of these results.

**Table 4:** Model averaging results presented as estimates and 95% confidence intervals

	Standardized variables			Untransformed variables		
Factor	Estimate	95% Confidence interval		Estimate	95% Confidence interval	
Intercept	20.26	18.20	22.33	58.68	41.86	75.50
Age (juvenile 1)	−2.39	−5.89	1.11	−2.39	−5.89	1.11
**Age (juvenile 2)**	**−4.23**	**−6.56**	**−1.90**	**−4.23**	**−6.56**	**−1.90**
Age (Sub-adult)	−1.91	−3.99	0.17	−1.91	−3.99	0.17
**Sex (male)**	**2.08**	**0.88**	**3.28**	5.14	−4.25	14.53
Season (spring)	1.24	−0.73	3.22	1.24	−0.73	3.22
Season (summer)	−1.43	−3.31	0.45	−1.43	−3.31	0.45
Season (winter)	0.92	−1.39	3.22	0.92	−1.39	3.22
**Mongoose**	**−1.35**	**−2.69**	**−0.01**	**−0.26**	**−0.51**	**−2.3e** ^**−3**^
Rabbit	1.19	−0.05	2.42	0.01	−4.0e^−4^	0.02
**Body condition (BCS)**	**−1.76**	**−3.09**	**−0.43**	**−2.8e** ^**−3**^	**−4.9e** ^**−3**^	**−7.0e** ^**−4**^
Human population	1.22	−0.18	2.62	0.01	−9.0e^−4^	0.01
**Body size (STL)**	**−5.20**	**−7.56**	**−2.84**	**−0.21**	**−0.34**	**−0.09**
**Storage**	**−3.65**	**−5.40**	**−1.90**	**−0.01**	**−0.01**	**−4.3e** ^**−3**^
**Distance to Tagus River**	**−1.76**	**−3.26**	**−0.26**	**−0.03**	**−0.06**	**−4.5e** ^**−3**^
Sex × body size (STL)	−1.74	−4.10	0.62	−0.08	−0.18	0.03
Region (historic)	−0.78	−3.28	1.72	−0.78	−3.28	1.72

Legend: estimates are presented for model averaging using input variables standardized on two standard deviations following Gelman (2008) and using untransformed input variables. Confidence intervals not including zero are highlighted in bold as variables are considered to significantly influence hGC concentration.

European rabbit and Egyptian mongoose harvest data were included in our model as proxies of relative prey availability and relative conspecific abundance, respectively. We expected hGC to decrease with relative prey availability and to increase with conspecific density. However, our results showed the opposite relation in both cases, with hGC increasing with prey availability and decreasing with mongoose abundance. The European rabbit is the Egyptian mongoose’s main prey species, accounting for 28% of ingested biomass in this specific population (Bandeira *et al.*, 2018). Food scarcity has been associated with increases in GCs in some species (Bryan *et al.*, 2013, 2014; Riechert *et al.*, 2014; Barrett *et al.*, 2015) but in others no effect was detected (Van Meter *et al.*, 2009; Rector *et al.*, 2012; Burstahler *et al.*, 2019).

An equally surprising result was the negative effect of relative mongoose abundance on hGC. It is not clear whether high population density always leads to an increased stress response. Studies linking the GCs to conspecific density and intraspecific competition in aquatic (Leatherland, 1993; Glennemeier and Denver, 2002; Bolasina *et al.*, 2006; Ramsay *et al.*, 2006; Teixeira *et al.*, 2012) and social (Hawley *et al.*, 2006; Eggermann *et al.*, 2013) species have shown inconsistent trends. In a non-social and aggressively territorial species like the Egyptian mongoose (Palomares and Delibes, 1993), the increased frequency of antagonistic social interactions is expected to result in increased HPA-axis activity at high population densities (Creel *et al.*, 2013), especially during the breeding season. While a possible explanation is that aggressive interactions driven by territorial behaviour cause only transient stress responses that might not be reflected in hGC, the limitations of our harvest data on these results cannot be ignored. Firstly, hunting yields might be more reflective of removal of individuals than indicators of abundance. Lower hGC levels could be a consequence of the continuous mongoose removal through hunting, potentially alleviating territorial competition. Secondly, hunting activities can cause stress (Bryan *et al.*, 2015; Vilela *et al.*, 2020). The increasing number of rabbits removed by hunting activities could lead to an increased stress response in mongooses in those areas either by acting as competition for resources or by causing direct disturbance to mongoose. Rabbit hunting often involves the use of firearms and hunting dogs, while mongoose captures are usually undertaken by trapping (Ministério da Agricultura, Desenvolvimento Rural e Pescas, 2000). This difference in hunting methods could explain an increase in hCG with increasing rabbit harvest numbers. Finally, hunting data have no correction for sampling effort, which can bias abundance estimations. Even when a catch-per-unit-effort metric is used, catch data may overestimate abundance (Harley *et al.*, 2001). In the specific case of the rabbit, discrepancies have been reported between abundance estimates using hunting data and field data (Ferreira *et al.*, 2010). Therefore, while harvest data might provide a good metric for the assessment of the physiological impact of hunting activities on the Egyptian mongoose, caution is necessary when interpreting them as indicators of abundance.

We found no support for our prediction that larger areas of Mediterranean shrub and agro-forestry habitats within each grid cell would be associated with lower levels of hGCs. This result could be due to the mongooses’ behavioural and dietary plasticity or alternatively by the dependence on resource-rich favourable habitat in the core areas of its territory that is not necessarily proportional to the size of the home range of each individual (Streicher *et al.*, 2020) or the area of favourable habitat available in each grid cell. Animal movement and high-resolution landscape data would be required to further analyse the relation between hGC and favourable habitat in the species.

## Conclusion

In the Anthropocene, species distributions are changing at an unprecedented rate. A small number of wild species have expanded their ranges, contradicting the general trend of decline. The study of these exceptions can help predict future species distributions. This study is the first to examine the relation between GCs and range expansion in mammals and uses a long-term measure of GC levels that is less subject to the short-term influence of environmental variables. The results show a decrease in hGC levels towards the expansion front, suggesting that the species’ expansion is unlikely to be limited by mechanisms related to- or mediated by the physiological stress response.

## Supplementary material

Supplementary material is available at *Conservation Physiology* online.

## Funding

This research was supported by the Leibniz Institute for Zoo and Wildlife Research, Berlin, Germany. V.B. and C.F. were supported by national funds through Fundação para a Ciência e a Tecnologia and European funds through the Programa Operacional Temático Factores de Competitividade and European Regional Development Fund by co-funding through the project ‘Genetic assessment of a successful invasion: Population genetics of the Egyptian mongoose (*Herpestes ichneumon*) in Portugal’ (PTDC/BIA-BEC/104401/2008) and by Fundação para a Ciência e a Tecnologia/Ministério da Ciência, Tecnologia e Ensino Superior for the financial support to CESAM (UIDP/50017/2020 + UIDB/50017/2020), through national funds.

## Supplementary Material

Table_S1_coab050
